# Bioinformatic Challenges in Clinical Diagnostic Application of Targeted Next Generation Sequencing: Experience from Pheochromocytoma

**DOI:** 10.1371/journal.pone.0133210

**Published:** 2015-07-31

**Authors:** Joakim Crona, Viktor Ljungström, Staffan Welin, Martin K. Walz, Per Hellman, Peyman Björklund

**Affiliations:** 1 Department of Surgical Sciences, Uppsala University, SE-75185, Uppsala, Sweden; 2 Department of Immunology, Genetics and Pathology, Uppsala University, SE-75185, Uppsala, Sweden; 3 Departments of Medical Sciences, Uppsala University, SE-75185, Uppsala, Sweden; 4 Department for Surgery and Centre of Minimal Invasive Surgery, Kliniken Essen-Mitte, Academic Teaching Hospital of the University of Duisburg-Essen, DE-45136 Essen, Germany; Huazhong University of Science and Technology, CHINA

## Abstract

**Background:**

Recent studies have demonstrated equal quality of targeted next generation sequencing (NGS) compared to Sanger Sequencing. Whereas these novel sequencing processes have a validated robust performance, choice of enrichment method and different available bioinformatic software as reliable analysis tool needs to be further investigated in a diagnostic setting.

**Methods:**

DNA from 21 patients with genetic variants in *SDHB*, *VHL*, *EPAS1*, *RET*, (*n*=17) or clinical criteria of NF1 syndrome (*n*=4) were included. Targeted NGS was performed using Truseq custom amplicon enrichment sequenced on an Illumina MiSEQ instrument. Results were analysed in parallel using three different bioinformatics pipelines; (1) Commercially available MiSEQ Reporter, fully automatized and integrated software, (2) CLC Genomics Workbench, graphical interface based software, also commercially available, and ICP (3) an in-house scripted custom bioinformatic tool.

**Results:**

A tenfold read coverage was achieved in between 95-98% of targeted bases. All workflows had alignment of reads to *SDHA* and *NF1* pseudogenes. Compared to Sanger sequencing, variant calling revealed a sensitivity ranging from 83 to 100% and a specificity of 99.9-100%. Only MiSEQ reporter identified all pathogenic variants in both sequencing runs.

**Conclusions:**

We conclude that targeted next generation sequencing have equal quality compared to Sanger sequencing. Enrichment specificity and the bioinformatic performance need to be carefully assessed in a diagnostic setting. As acceptable accuracy was noted for a fully automated bioinformatic workflow, we suggest that processing of NGS data could be performed without expert bioinformatics skills utilizing already existing commercially available bioinformatics tools.

## Introduction

About 35% of Pheochromocytoma (PCC) and Paraganglioma (PGL) patients present with a pathogenic germline or mosaic variant conferring susceptibility to the disease [1]. A total of eighteen genes have been described as disease causing and these loci constitute 25 kilo base pairs spanning through 196 different exons [2–15]. Given the performance of available methods for diagnostic genetic screening, a comprehensive analysis including all PCC and PGL disease causing loci is both extensively recourse demanding and time consuming [16]. Instead, selected fragments are prioritized for diagnostic analysis guided by patient phenotype and/or immunohistochemistry [17]. Such selective screening may have reduced sensitivity and not all patients will be referred for a genetic consultation [18].

Recent publications described next generation sequencing (NGS) workflows for the analysis of genes conferring susceptibility to PCC and PGL [19–21]. Rattenberry et al. suggested near equal quality to Sanger sequencing (SS) and a significant reduction in both cost and time consumption [19]. Similar performance of diagnostic targeted NGS has been reported by an accumulating number of observations in other disease contexts that have used multiple different enrichment assays and sequencing platforms [19, 22–25]. While the robustness of basecalling has been demonstrated across principally different technologies, the performance of multiplexed targeted enrichment and bioinformatic processing have not been thoroughly validated in diagnostic application [26, 27]. Current guidelines for the diagnostic use of next generation sequencing state that the validity of the selected bioinformatic software needs to be ensured by the local investigator [28]. Hence, the local laboratory should select, validate and maintain a robust bioinformatics pipeline, a process that will require trained and experienced personnel. These investments and the running costs of bioinformatic processing will inevitably increase cost of targeted NGS and has been predicted to exceed the total cost of sequencing and enrichment [29].

As current methods impose restrictions in the genetic screening of PCC and PGL patients we initiated a study investigating the use of targeted DNA enrichment, sequenced on a next generation bench top sequencer, utilizing three different bioinformatics tools and compared to results from traditional Sanger sequencing.

## Methods

### Patients

This was a retrospective study including 21 patients with PCC recruited at two different centres; Uppsala university hospital, Uppsala, Sweden and Kliniken Essen-Mitte, Academic Hospital of the University of Duisburg-Essen, Essen, Germany. Inclusion criteria were; (1) histopathological diagnosis of PCC or PGL; and (2) a confirmed pathogenic genetic mutation and/or clinical criteria of a PCC or PGL syndrome diagnosed by a specialist in clinical genetics. Of the included patients 17 had a described variant in *SDHB*, *VHL*, *EPAS1* or *RET* and four had clinical criteria of NF1 syndrome [30]. For the Uppsala cohort sequencing data and the presented variants have been partially exploited in previous studies [30–32].

### Ethical statement

Samples were obtained from Uppsala Biobank, Endocrine tumour collection (Ethical approval 00-128/ 3.15.2000, Local ethical vetting board in Uppsala (Regionala etikprövningsnämnden i Uppsala)). The study was approved by the regional ethical review board in Uppsala (12-422/ 11.21.2012 and 05-198/ 8.10.2005, Local ethical vetting board in Uppsala (Regionala etikprövningsnämnden i Uppsala)). Written informed consent was obtained from the individual patients. All patients were above 18 years of age at the time of inclusion.

### DNA and RNA extraction

Genomic DNA from available tissue samples were extracted using DNeasy Blood & Tissue Kit (Qiagen, Hilden, Germany) as previously described [33]. DNA quality and concentrations were assessed using Nanodrop spectrophotometer (ThermoFischer Scientific, MA, USA) and Qubit Flourometer (Invitrogen, CA, USA). Sample inclusion criteria were a 260/280 spectrums ratio of >1.8 and double strand DNA concentrations above 5ng/μl. RNA was extracted using AllPrep DNA/RNA kit (Qiagen) and was subjected to reverse transcription using RevertAid First Strand cDNA Synthesis Kit (Thermo Scientific, Waltham, MA, USA).

### Sanger Sequencing characterization

All included samples had been screened with automated Sanger Sequencing (SS, Beckman Coulter Genomics, Takeley, UK) for a comprehensive panel of 9 disease causing genes to be utilized as negative and positive control (cumulative size 5683 basepairs); *SDHB*, *SDHC*, *SDHD*, *SDHAF2*, *VHL*, *EPAS1* (Exons 9 and 12) *RET* (exons 10–11 and 13–16), *TMEM127* and *MAX* as previously described [30].

### Targeted genomic capture

A Truseq Custom Amplicon (Illumina Inc, San Diego, CA, USA) targeted capture and paired end library kit was designed using Illumina Design Studio (Version 2012-09-13, http://designstudio.illumina.com). All coding sequences of eleven established PCC and PGL disease causing genes; *SDHA*, *SDHB*, *SDHC*, *SDHD*, *SDHAF2*, *VHL*, *EPAS1*, *NF1*, *RET* (exons 8, 10–11 and 13–16), *TMEM127* and *MAX* were selected. A novel disease causing gene, *H-RAS* was selected for enrichment but excluded from further analysis because of the lack of clinical relevance for investigating this loci (Table 1). In order to be able to detect variants causing alternative splicing, coordinates were extended with a padding of 10 base pairs at intron-exon boundaries. Coordinates were obtained from the human reference sequence HG19 and the cumulative target size was 24,293 base pairs. The final TruSeq Custom Amplicon design constituted 331 amplicons having a median size of 177 bases. The *in silico* amplicon coverage was >99% with a total gap distance of 57 bases located in a region with homologous sequences; *SDHC* exon 2.

**Table 1:** Targeted enrichment, included genomic regions.

Gene	Chr	Gene Cordinates	Exons	Cumulative amplicon size, basepairs
*SDHA*	5	218356–256815	15	6475
*SDHB*	1	17345217–17380665	8	2625
*SDHC*	1	161284047–161332984	6	1925
*SDHD*	11	111957497–111990353	4	1400
*SDHAF2*	11	61197514–61215001	5	1750
*VHL*	3	10182692–10193904	3	1400
*EPAS1*	2	46520806–46613836	16	7700
*RET*	10	43572475–43625799	20	2800
*NF1*	17	29421945–29709134	58	27300
*TMEM127*	2	46520806–46613836	4	2100
*MAX*	14	65472892–65569413	5	1575
*H-RAS*	11	532243–535550	6	1050

Selected genomic regions for targeted enrichment utilized in the TruSeq Custom Amplicon assay. Sequences annotated as protein coding were selected in *SDHA*, *SDHB*, *SDHC*, *SDHD*, *SDHAF2*, *VHL*, *EPAS1*, *RET*, *NF1*, *TMEM127*, *MAX* and *H-RAS*. In *RET* exons 8,10–11,13–16 were selected. The design was extended with 10 base pairs at exon-intron junctions.

### Library preparation and MiSEQ sequencing

Truseq custom amplicon sample kit (Cat. No. FC-130-1001, FC-130-1006, FC-130-1007, Illumina Inc) for targeted capture and library preparation were prepared from 250ng of double stranded DNA accordingly to manufacturers instructions (Part# 15027983). Briefly, upstream and downstream oligonucleotides were hybridized to genomic DNA and unbound oligonucleotides were washed away using ELM3, SW1 and UB1 washing reagents. This was followed by an extension ligation process that connected hybridized upstream and downstream oligonucleotides by using DNA ligase. Extension-ligation products were amplified by PCR and fitted with index adaptor sequences for sample multiplexing using the TruSeq Custom Amplicon Index Kit (Cat. No. FC-130-1003, Illumina Inc). The PCR product was purified from reaction components using AMPure XP beads (Illumina Inc) and selected test samples were run on a 4% agarose gel to confirm successful library preparation. Each library sample underwent quantity normalization by LNA1/LNB1 beads (Illumina Inc) and all 21 samples were pooled together with 74 samples of other origin into a single suspension. Generated paired end (2x150bp) libraries were subjected to two independent sequencing runs on the Illumina MiSEQ platform (Illumina Inc). Sequencing was performed at the university core facility (http://molmed.medsci.uu.se/SNP+SEQ+Technology+Platform/) as instructed in awere automatically demultiplexed by MiSEQ integrated software and results written to .FASTQ files.

### Read mapping and variant calling

**Fig 1:**
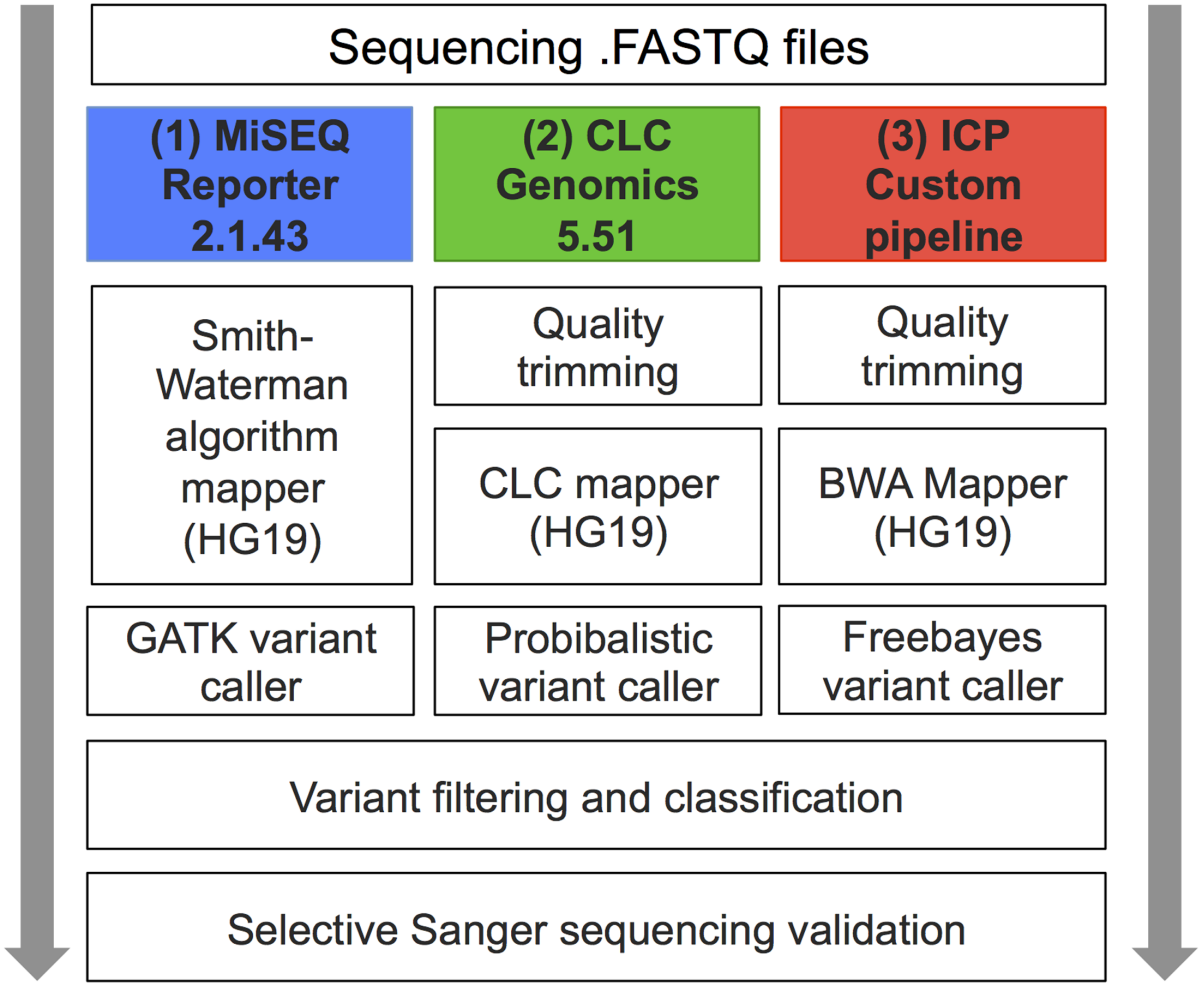
Overview of Bioinformatic workflows. Schematic description of the utilized workflows MiSEQ Reporter 2.1.43 (Illumina inc), CLC Genomics Workbench 5.51 (CLCbio) and the in-house custom pipeline (ICP).

Generated sequences were processed in-house using three different bioinformatics software workflows (Fig 1): (1) automated pipeline, MiSEQ reporter v2.1.43 (Illumina Inc); (2) semi automated pipeline, CLC Genomics Workbench 5.51 (CLC bio, Aarhus, Denmark); and (3) ICP a in-house custom pipeline constituting of multiple different publicly available software packages as described below. Default settings were used in workflow (1). In workflows (2) and (3) the settings of the variant callers were optimized by using the known pathogenic genetic variants as reference material. The probability and variant allele thresholds were lowered as to achieve the maximum detection of true positive variants in CLC and Freebayes respectively.

In workflow 1 (MSR) a manifest file stating the sequence of hybridizing oligos and the coordinates of attempted amplicons was downloaded from the manufacturer (ww.illumina.com) and uploaded into the MiSEQ reporter 2.1.43 software as instructed (Part# 15038604). Briefly, reads were matched to the sequence coordinates overlapping those of the included truseq custom amplicon probes. The reads were subsequently aligned to the reference sequence (human reference sequence HG19) using Smith-Waterman algorithm with default settings. Reads that were not matched to probes or having multiple alignments were discarded. Variant calling was performed using a variant caller from the Genome Analysis Toolkit (GATK) with default settings [34]. Generated .BAM and .VCF files were exported for annotation, filtering and further analysis.

In workflow 2 (CLC) the generated .FASTQ files were imported into CLC Genomics Workbench 5.51 and processed with the following steps: 1; Sequence quality trimming based on Phred quality scores and removal of ambiguous base-calls, 2; Read Mapping (human reference sequence HG19) with <3 mismatches / 100bp for alignment, 3; variant calling using a probabilistic based algorithm (Probabilistic Variant Detection Plug-in Manual, clcbio.com) reporting variants having a probability of above 90%. The variant caller was set to exclude variants available in broken read pairs, unspecific read alignments as well as variants exclusively in found forward/reverse reads.

In workflow 3 (ICP) read mapping was performed using the Burrows Wheeler Alignment tool (BWA) with default parameters [35]. The generated SAM files were converted to BAM format, sorted and indexed using Samtools [36]. Variant calling was performed using Freebayes [37]. Variants with a minimum coverage of 30 reads and allele frequency of > 10% were reported. Generated .BAM and .VCF files were exported for annotation, filtering and further analysis.

Generated .BAM files from workflows (1), (2) and (3) were inported into CLC Genomics Workbench 5.51 and analysed for sequence coverage and depth. Targeted bases were defined as the protein coding sequences of the included 11 genes with clinical relevance (excluding *H-RAS*) cumulative size 18324. Generated .VCF files from workflows (1), (2) and (3) were filtered and annotated in CLC Genomics Workbench 5.51. Step 1; removal of synonymous variants without a probable splice site effect. Step 2; the remaining variants were annotated for overlapping information in selected genetic databases; Single Nucleotide Polymorphism database (dbSNP) build 137, Catalogue of Somatic Mutations in Cancer (COSMIC) [38], database of Human Gene Mutation Data (HGMD) [39] and Leiden Open (source) Variant Database (LOVD).The impact of non-synonymous amino acid substitution was assessed *in silico* using Polymorfism Phenotyping v2 (Polyphen2) [40] and Sorting Intolerant from Tolerant (SIFT) [41]. Overlapping variants were analysed with a custom R script. Variants were classified as Pathogenic, Unknown (Variant of Unknown Significance, VUS) or Polymorphism and selected entries were validated with Sanger sequencing. Primer sequences can be obtained by request.

## Results

A summary of patient characteristics and discovered genetic variants is presented in Table 2. Samples generated a total of 4961465 (run 01) and 4914971 (run 02) reads. The mean sequencing output per sample was 236380 (range 163818–291262) for run 01 and 230795 (range 163548–302238) for run 02. Results will be presented in parallel for the three workflows; MiSEQ Reporter 2.1.43 (MSR), CLC Genomics Workbench 5.51 (CLC) and the in-house custom pipeline (ICP).

**Table 2:** Detected mutations and patient characteristics

Patient no.	Age, years	Gender	Syndrome diagnosis	Genotyping
1	15	Male	PGL4	*SDHB* p.Arg90*
2	48	Male	VHL	*VHL* p.Tyr98His
3	26	Male	VHL	*VHL* p.Arg107Ser
4	66	Female	VHL	*VHL* p.Arg161Gln
5	11	Male	VHL	*VHL* p.Val170Gly
6	46	Male	VHL	*VHL* p.*214Gly
7	64	Female	-	*EPAS1* p.Leu529Pro (S)
8	81	Female	-	*EPAS1* p.Ala530Val (S)
9	27	Female	MEN2A	*RET* p.Cys609Ser
10	36	Female	MEN2A	*RET* p.Cys611Tyr
11	29	Female	MEN2A	*RET* p.Cys634Arg
12	29	Male	MEN2A	*RET* p.Cys634Gly
13	30	Female	MEN2A	*RET* p.Cys634Tyr
14	27	Female	MEN2A	*RET* p.Tyr791Phe
15	65	Male	MEN2A	*RET* p.Met804Val
16	34	Male	MEN2B	*RET* p.Met918Leu
17	18	Female	MEN2B	*RET* p.Met918Leu
18	50	Male	NF1	*NF1* p.Arg1241*
19	44	Male	NF1	*NF1* p.Trp2494*
20	57	Female	NF1	*NF1* c.288+1G>T
21	56	Female	NF1	*NF1* p.Lys1844*

Summary of patient characteristics, syndrome criteria and discovered genetic variants. PGL4; Familial Paraganglioma type 4, VHL; Von Hippel Lindau, MEN2; Multiple Endocrine Neoplasia Type 2; and NF1, Neurofibromatosis type 1. (S); Not detected in DNA from peripheral blood.

* indicates stop codon.

### Read mapping

**Fig 2:**
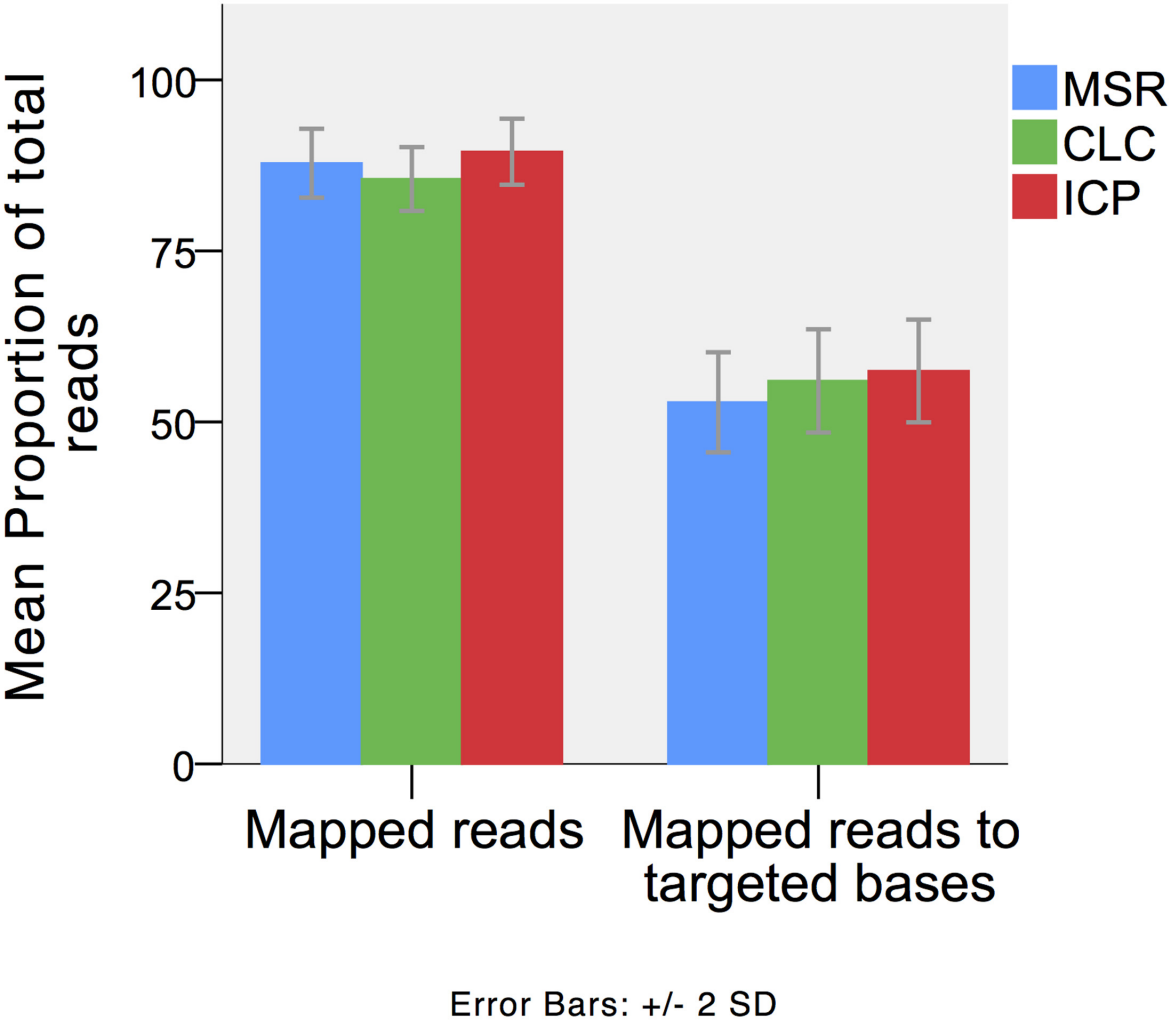
Read mapping. Results from the three different workflows; MSR; MiSEQ Reporter 2.1.43 (Illumina Inc), CLC; CLC Genomics Workbench 5.51 (CLCbio); and ICP, in-house custom pipeline. The proportion of reads mapped to the reference sequence and targeted bases respectively.

**Fig 3:**
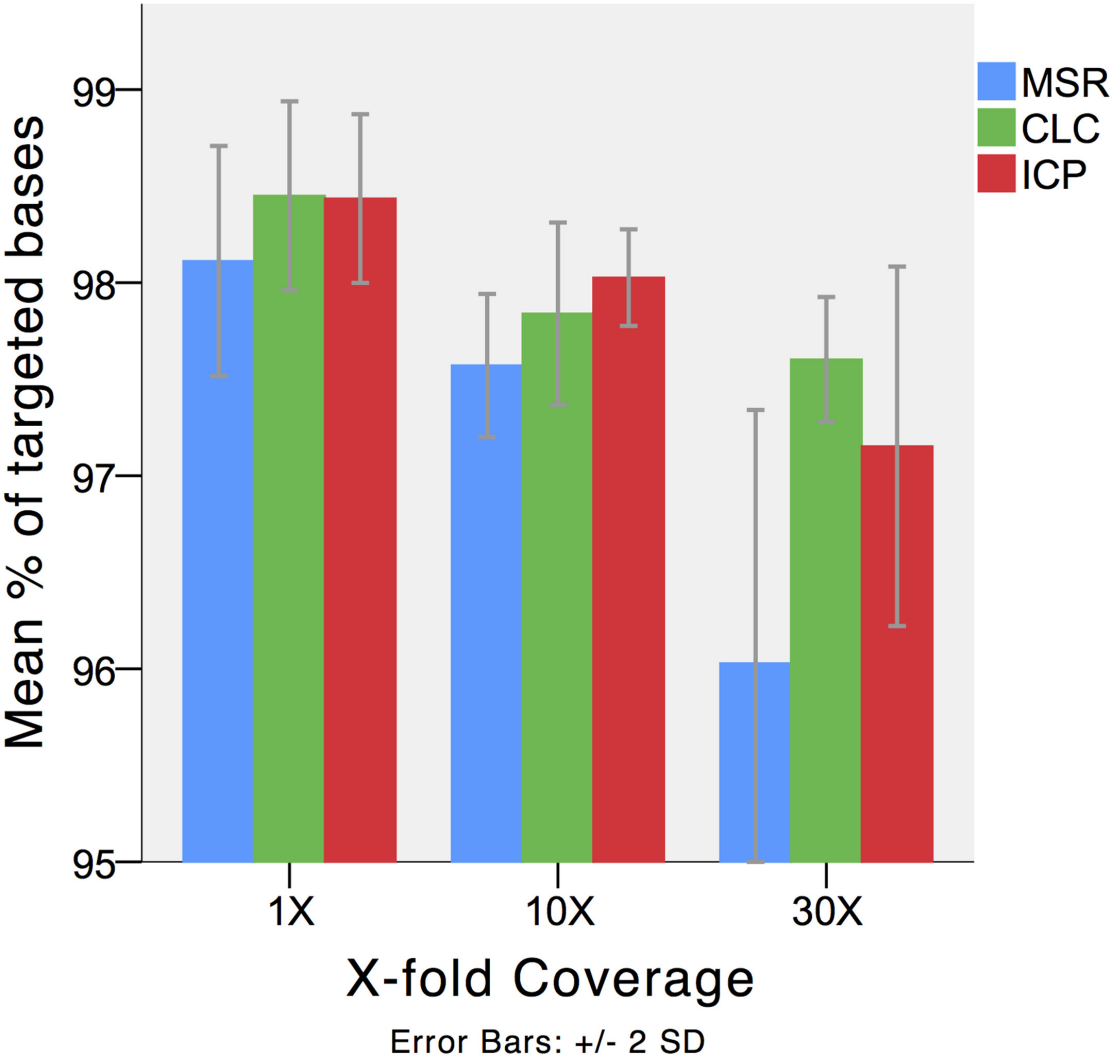
Coverage. Results from the three different workflows; MSR; MiSEQ Reporter 2.1.43 (Illumina Inc), CLC; CLC Genomics Workbench 5.51 (CLCbio); and ICP, in-house custom pipeline. The proportion of targeted reads that achieved X-fold coverage. Error bars represent +/- 2 standard deviations.

Results from read mapping are presented in detail in Table 3 and Figs 2 and 3. Alignment resulted in a mean 87.8% (MSR), 85.5% (CLC) and 89.5% (ICP) of reads mapped to the human reference sequence. The specificities for targeted regions were 52,9% (MSR), 57% (CLC) and 58% (ICP). A total of 97.6% (MSR), 97.8% (CLC) and 98.0% (ICP) of targeted bases had a 10 fold sequencing depth. A 30-fold sequencing depth were achieved in 96.1% (MSR), 97.6% (CLC) and 97.15% (ICP) of the targeted bases. The mean cumulative sizes of targeted regions without mapped reads were 114 (MSR), 91 (CLC) and 92 (ICP) base pairs. The regions without aligned reads were located in *SDHC* exon 2, *SDHAF2* exon 2 and *NF1* exon 57. Alignments of reads to sequences outside the targeted regions were detected in all three pipelines included significant alignment to *SDHA* (*SDHAP1*, *SDHAP2*) and *NF1* (*NF1P1*, *NF1P3*, *NF1P6* and *NF1P8*) psueodogenes.

**Table 3:** Read Mapping

				% X-fold coverage at targeted regions		
	Mapped reads to HG19 reference, mean % (range)	Mapped reads to targeted regions, mean % (range)	Read coverage, mean (Range)	1X, mean (range)	10X, mean (range)	30X, mean (range)
**MiSEQ Reporter 2.1.43**						
Run01	89.4 (82–92.2)	54 (48.3–64.1)	414 (284–565)	98.1 (97.6–98.4)	97.6 (97.4–98)	95.9 (94.6–96.9)
Run02	86.3 (82.1–88.5)	51.7 (46.6–61.4)	407 (283–571)	98.2 (97.9–98.5)	97.6 (97.3–98.3)	96.2 (94.6–97.3)
**CLC Genomics Workbench 5.51**						
Run01	86.9 (82.6–92)	56.9 (50.5–67.2)	552 (377–738)	98.4 (98–98.7)	97.9 (97.7–98.4)	97,6 (97,3–97,7)
Run02	84.1 (80–89.1)	55.1 (49.3–64.7)	526 (357–735)	98.5 (98–98.7)	97.8 (97.6–98.7)	97,6 (97,2–98)
**In-house custom pipeline**						
Run01	89.6 (82–92.2)	58 (51.5–68)	519 (357–699)	98.4 (98–98.7)	98 (97.9–98.3)	97 (96.2–97.6)
Run02	89.4 (82.3–92.4)	56.9 (50.4–66.1)	517 (355–711)	98.5 (98.2–98.7)	98.1 (97.8–98.5)	97.3 (96.3–98)

Results from read mapping. Data presented from three bioinformatics workflows; MiSEQ Reporter 2.1.43 (Smith Waterman algorithm mapper), CLC Genomics Workbench 5.51 (default CLC mapper) and in-house custom pipeline (Burrows Wheeler Alignment tool).

### Variant calling

**Fig 4:**
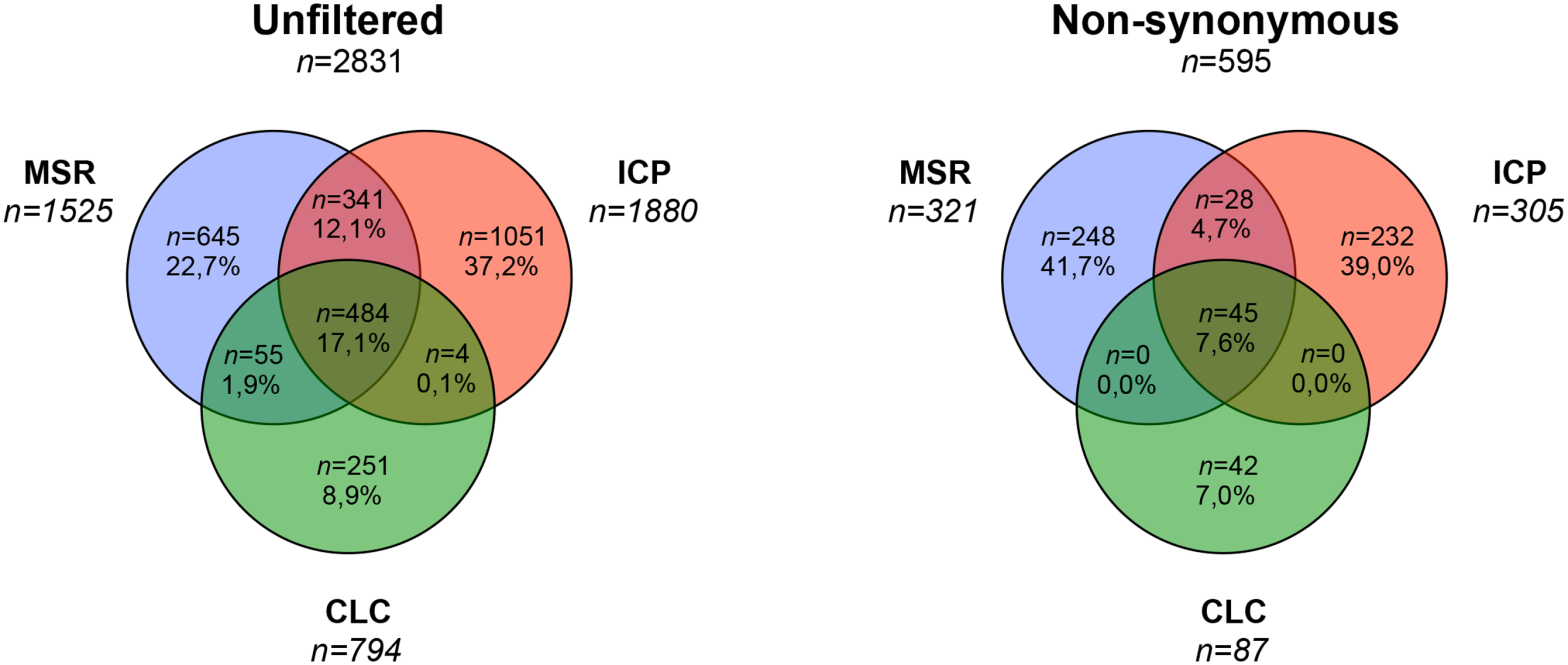
Venn diagram of overlapping variants between the three workflows, total (all variants available at bases annotated for the 11 included genes) and non synonymous remaining variants after filtering synonymous variants with no calculated splice site disruption. MSR; MiSEQ Reporter, CLC; CLC Genomics Workbench, and ICP; In-house custom pipeline.

**Fig 5:**
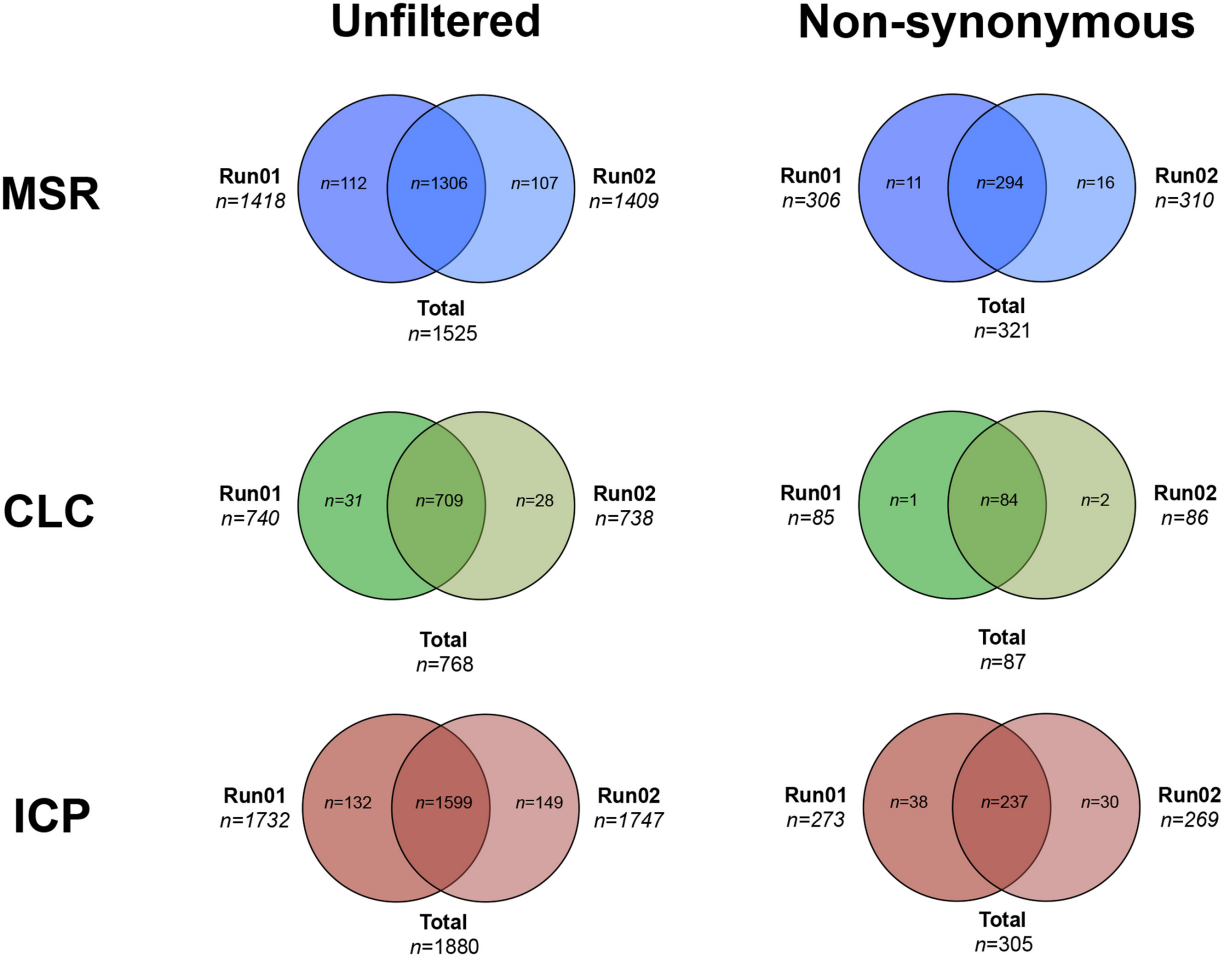
Venn diagram of overlapping variants between the two sequencing runs, total (all variants available at bases annotated for the 11 included genes) and non synonymous remaining variants after filtering synonymous variants with no calculated splice site disruption. MSR; MiSEQ Reporter, CLC; CLC Genomics Workbench, and ICP; In-house custom pipeline.

Results from variant calling are presented in detail in Table 4 and Figs 4 and 5. Variant calling revealed a total of 1525 (MSR, Run01; 1418, Run02; 1409), 768 (CLC Run01; 740, Run02; 738) and 1880 (ICP Run01; 1732, Run02; 1747) variants located in the targeted genes. Subsequent filtering of synonymous variants with no probable splice effect resulted in 321 (MSR), 87 (CLC) and 305 (ICP) remaining variants. Out of 47 variants detected by Sanger sequencing, MSR detected all 47 variants in both sequencing runs, CLC detected 39 (run01) and 40 (run02) variants and ICP detected 42 (run01) and 43 (run02) variants. Results from variant calling corresponded to a sensitivity of 100/100% (Run01/02 MSR), 82,9/85,1% (Run 01/02 CLC) and 89,4/91,4% (Run01/02 ICP). CLC did not detect *VHL* p.Tyr98His (run01, patient 3), *EPAS1* p.Leu529Pro (Run 01 and 02, patient 8), *RET* p.Cys611Tyr (Run01, patient 11) and *NF1* p.Arg1241* (run01, patient 19). *SDHA* p.Tyr629Phe was not detected by CLC or freebayse in any of the sequencing runs. The specificity was >99.99% for MSR and ICP while CLC had a perfect 100% specificity (Table 5). The number of false positive variants could be reduced by removal of variants not available in both sequencing runs in the MSR and ICP workflows. In total 17% of variants were reported among all workflows and about 60% were specific to a single workflow.

**Table 4:** Variant calling

	Total variants	Variants per sample, mean (range)	Substitutions per sample, mean (range)	INDELs per sample, mean (range)
**MiSEQ Reporter 2.1.43**				
Run01	1418	75 (30–92)	63 (23–80)	12 (7–15)
Run02	1409	75 (60–96)	62 (48–82)	12 (6–15)
**CLC Genomics Workbench 5.51**				
Run01	740	34 (22–52)	28 (17–44)	6 (3–9)
Run02	738	32 (20–51)	27 (17–42)	5 (3–9)
**In-house custom pipeline**				
Run01	1732	85 (40–102)	73 (33–90)	12 (7–15)
Run02	1747	85 (70–106)	72 (58–92)	12 (6–15)

Results from variant calling in the three workflows MiSEQ Reporter 2.1.43 (GATK variant caller), CLC Genomics Workbench 5.51 (Probalistic variant caller) and the in-house custom pipeline (Freebayse variant caller). Results are presented as total number of variants in targeted regions and variants per sample.

**Table 5:** Sensitivity and specificity compared to Sanger Sequencing.

	True pos, *n*	True neg, *n*	False pos, *n*	False neg, *n*	Sensitivity, %	Specificity, %
MiSEQ Reporter 2.1.43						
Run01	47	119197	99	0	100	>99.99
Run02	47	119195	101	0	100	>99.99
Filtered, merged Runs 01+02	47	119190	94	0	100	>99.99
CLC Genomics Workbench 5.51						
Run01	39	119296	0	8	82.9	100
Run02	40	119296	0	7	85.1	100
Filtered, merged Runs 01+02	36	119296	0	11	76.7	100
In-house Custom pipeline						
Run01	42	119284	7	5	89.4	>99.99
Run02	43	119288	4	4	91.4	>99.99
Filtered, merged Runs 01+02	42	119285	4	7	87.2	>99.99

Sensitivity of targeted next generation sequencing compared to current golden standard (automated Sanger sequencing) covering 5683 basepairs. Results are presented separately for both sequencing runs as well. Filtered and merged results incudes only variants available in both sequencing runs. Pos; positive, neg; negative.

### Variant classification

**Fig 6:**
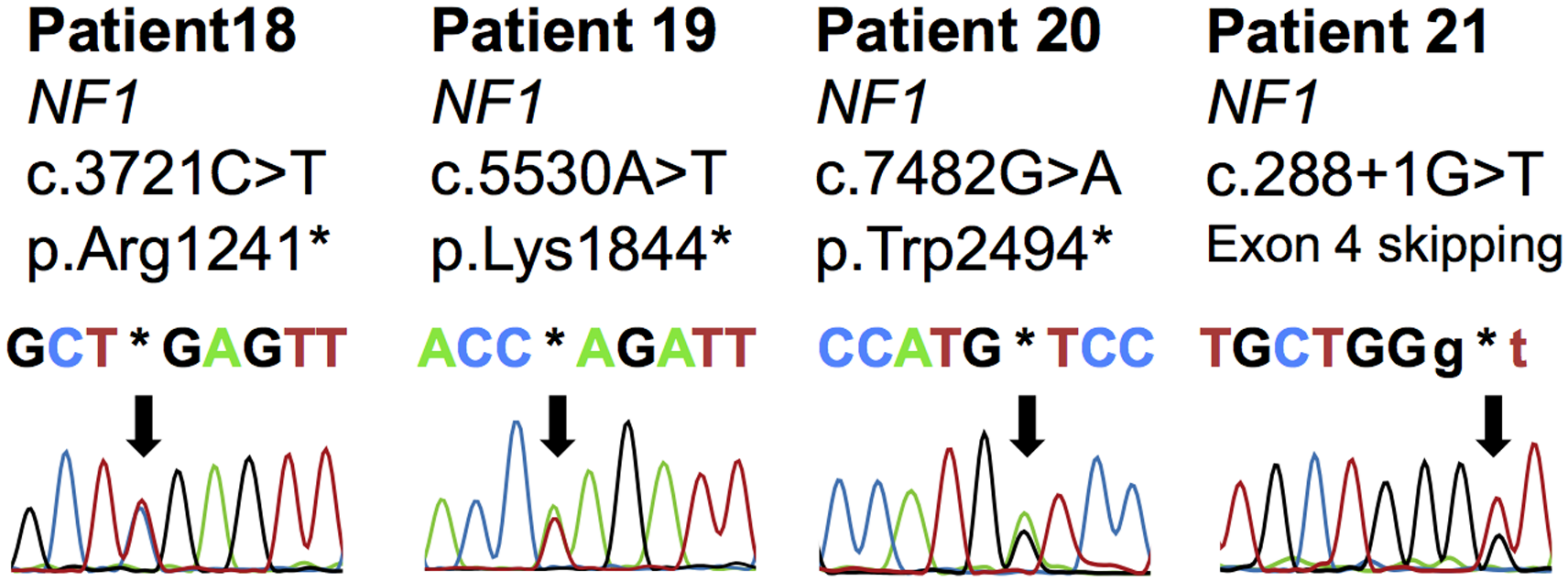
Detected NF1 mutations. Chromatograms from automated Sanger sequencing as displayed in CLC Genomics Workbench 5.51 (CLCbio). *NF1* variants available in germline DNA that were classified as pathogenic.

All pathogenic variants occurred in a non-concomitant fashion and all but one patient had a pathogenic variant in one of the investigated genes. One patient had a pathogenic germline mutation in *SDHB* (p.Arg90*). Five different pathogenic germline variants were confirmed in patients with clinical criteria of von Hippel Lindau syndrome (p.Tyr98His, p.Arg107Ser, p.Arg161Gln, p.Val170Gly and p.*214G). There were two pathogenic *EPAS1* variants (p.Leu529Pro and p.Ala530Val) that were detected in DNA from tumoral tissue, these variants were absent in DNA from peripheral blood. Eight patients with clinical criteria of MEN2 had pathogenic germline variants in *RET* (p.Cys609Ser, p.Cys611Tyr, p.Cys634Arg, p.Cys634Gly, p.Cys634Tyr, p.Met804Val, p.Met918Leu and p.Met918Leu). There were three nonsense variants detected in germline DNA from three different patients with clinical criteria of NF1 syndrome; p.Arg1241*, p.Lys1844* and p.Trp2494* (Fig 6). One additional patient with NF1 syndrome had a germline variant resulting in alternative splicing; c.288+1G>T. Sequencing of cDNA derived from peripheral blood revealed skipping of exon 4. These four variants in *NF1* were all classified as pathogenic. One patient had only variants of unknown significance including *RET* p.Tyr791Phe.

### Performance

Following implementation of the workflow and optimization of the bioinformatics workflow, the total throughput time for NGS was 7 days divided on 1 day for sample preparation and quality assessment, 2 days for sample enrichment and multiplexing, 1 day for MiSEQ sequencing and 1 day for bioinformatics processing and interpretation. Validation with Sanger sequencing may be estimated to an additional 7–14 days. In our laboratory we were able to reduce the cost of sequencing per exon from 6.5 USD to 0.56 USD for this experiment (S1 Table).

## Discussion

The present study validated an amplicon based next generation sequencing method for diagnostic re-sequencing of 11 genes (including *EPAS1* and *NF1*) associated with PCC and PGL tumours. To determine the robustness of bioinformatic processing, three principally different bioinformatics pipelines were compared. Only the fully automated and integrated software package reported all variants detected by Sanger Sequencing. We confirmed that targeted NGS have superior performance and comparable quality to Sanger Sequencing. Our results further suggest that the bioinformatics analysis needs to be carefully reviewed before clinical application and that the analysis can be performed using automated software.

The mapping process generated comparable results across the different workflows with about 85–90% and 55% of reads being mapped to the human reference and targeted sequences respectively. Several factors may contribute to this relatively low on target proportion. Multiple amplicons were located at intro/exon boundaries, and as a consequence a proportion of sequencing reads were located outside the protein coding sequences (targeted regions). Unspecific read alignment was also detected with reads mapped to *SDHA* and *NF1* pseudogenes as well as to genomic regions without annotation. The proportion of targeted sequences that achieved an appropriate read coverage was similar to previous studies that have identified regions with high GC content being difficult to amplify during enrichment and library preparation [21, 42]. Indeed several of the genes conferring susceptibility to PCC and PGL have high GC content and/or multiple pseudogenes that may complicate the design and interpretation of genetic testing and 100% coverage may be hard to achieve [19]. A potential impact of the unspecific amplification cannot be ruled out as a defined region in *NF1* exon 21 had a relatively high number of false positive variants. But as these findings occurred in a stochastic manner and the variants could be removed by subtracting variants not available in both sequencing runs. To reduce unspecific amplification, a high degree of flexibility regarding amplicon size and oligo location is warranted when designing the multiplex assay for genomic enrichment.

With regards to variant calling there was pronounced heterogeneity observed between the three workflows. Only a minority of the detected variants were shared between the three bioinformatics pipelines. Examining these variants that were not overlapping between the workflows were most often positioned outside protein coding regions in amplicon start/ends and often occurred in a high frequency of the included cases. Only one workflow managed to detect all variants within the reference panel; MSR. The MSR analysis was performed with default settings that achieved optimal processing despite the limited flexibility of the software. The ICP workflow failed to detect one pathogenic variant, located to *RET* codon 804, in one of the sequencing runs. This false negative was probably due to low coverage, a phenomenon that was detected in this region in all three workflows. CLC did not detect several variants despite extensive optimization with focus on sensitivity for pathogenic variants. Even so this workflow generated the lowest number of variants and had the highest specificity and it cannot be ruled out that the selected settings for variant calling was to stringent resulting in a lower sensitivity. The variability of generated variants among the different workflows is comparable to previous studies showing similar differences between NGS bioinformatic pipelines [43, 44].

The rationale to include workflows having a graphical interface and a high degree of automatization in diagnostic bioinformatics analysis would be potential cost reductions. This may be achieved through outsourcing of certain bioinformatics tasks to staff with intermediate computational skills. Due to the full integration of the MiSEQ reporter software, the total hands-on time was reduced to a few minutes and there was no time needed for optimization of the workflow. A graphical interface and high degree of automatization is shared by CLC that allows for a higher degree of flexibility in both read mapping and variant detection. However, the optimization process was long, as the workflow could not be tuned to report all variants detected by Sanger sequencing. The command-based workflow (ICP) had an intermediate profile both in regards of performance and total hands on time. Our results suggest that software with a graphical interface and a high degree of automatization may allow outsourcing of certain tasks to less experienced staff and could therefore be cost effective (with equal quality).

The momentum of NGS in a clinical setting was recently strengthened by demonstrating equal quality of generated results compared to SS [45]. A subsequent proof of principle study for the analysis of nine genes associated with PCC and PGL tumours suggested that targeted next generation sequencing would be beneficial with a 70% cost reduction and 66% increase in diagnostic yield compared to sanger sequencing [19]. Results from this study confirmed these specifications and were further able to screen the *NF1* gene and *EPAS1* for somatic mutations. Germline mutations in *NF1* and mosaic mutations in *EPAS1* have recently been found in apparently sporadic PCC patients, and would increase the diagnostic yield if in included into the analysis [21, 46, 47].

We conclude that targeted next generation sequencing has equal quality compared to Sanger sequencing. Enrichment specificity and the bioinformatic sensitivity need to be ensured in each clinical diagnostic application. As superior accuracy was noted for a fully automated bioinformatic workflow compared to two other bioinformatics tools, we suggest that handling of NGS data could be performed without expert bioinformatics skills utilizing commercially available software.

## Supporting Information

S1 Table Cost for bioinformatic workflows. * Given that sequencing provider provides the analysis.
(DOCX)
