# Supplementary material for: Bioinformatic Challenges in Clinical Diagnostic Application of Targeted Next Generation Sequencing: Experience from Pheochromocytoma
Source: PLoS One. 2015 Jul 31;10(7):e0133210. doi: 10.1371/journal.pone.0133210 (PMC4521794; doi:10.1371/journal.pone.0133210)
Supplement: S1 Table — * Given that sequencing provider provides the analysis. (DOCX) [file pone.0133210.s001.docx]

| **S1 Table. Costs of the respective workflows** | | | | |
| --- | --- | --- | --- | --- |
|  | **Investment** | **Running cost** | **Flexibility and adjustability** |  |
| MSR | None* | None* | Limited |  |
| CLC | Hardware, Software | Staff with limited training | Excellent |  |
| Academic | Hardware | Staff with computational training | Excellent |  |
|  |  |  |  |  |
| * Given that sequencing provider provides the analysis | | | | |
